# Four Cases of Surgical Kidney

**Published:** 1888-08

**Authors:** I. N. Danforth

**Affiliations:** Professor of Renal Diseases in The Woman’s Medical College


### FOUR CASES OF SURGICAL KIDNEY.

BY I. N. DANFORTH, A. M., M. D.,
Professor of Renal Diseases in The Woman's Medical College.

CASE I.—On the 18th of February, 1879, I was called to see Mrs. I., an Irish woman, aged thirty-eight, married, but without having borne children. She was sitting upon her bed, propped up by pillows, and partly supporting the weight of the upper half of the body upon her clenched fists. Her room was without a window; the atmosphere was saturated with the odor derived from a dirty kerosene lamp and from the exhalations from a livery stable immediately under her. Her face was pinched with agony, and as I entered the dark and fœtid room, she had a paroxysm of hysterical weeping. She soon regained her composure, however, and I then elicited the following history of her case: About five years prior to my visit, while under an unusual strain of hard work in a restaurant, she contracted acute cystitis, and suffered the usual symptoms, namely, incontinence, dysuria, hæmaturia, etc. After trying various domestic remedies and patent nostrums, she called in a practitioner of the "irregular" sort, who promised her a speedy cure. His treatment was both general and local, the latter being vigorous, to say the least. It was as follows: Introducing a steel sound into the bladder, he attached one pole of a galvanic battery thereto, while the other pole was placed over the bladder just above the pubes He then passed a current through her bladder for nearly half an hour. The effect was intense pain, with cystic tenesmus, hæmaturia, and immediate intensification of all her symptoms. From that time until I saw her, she was treated by various practitioners, and by every local and general means which the books suggested or ingenuity could devise. Nevertheless, in spite of treatment, or in consequence of it, she steadily grew worse until she had reached the miserable condition in which I found her. She was thin, pale, and sallow, and very much emaciated. Her digestion was exceedingly poor; her bowels obstinately constipated in spite of the almost constant pelvic tenesmus, and she was troubled with frequent attacks of nausea and vomiting. Between trying to urinate and trying to resist urination, she was kept busy night and day, so that she got scarcely any restful sleep. Her urine was rather scanty, intensely alkaline, strongly ammoniacal, and emitted a very offensive odor. It was heavily loaded with pus, phosphates, and sodic urate; it also contained a little albumen, and a large amount of mucus. The microscope showed pus-corpuscles in every stage of growth and decay, epithelial cells in large numbers from all parts of the urinary tract, considerable amorphous granular matter, a few large short casts from the straight tubes, much eroded from maceration in alkaline urine, and crystals of the saline constituents already mentioned. At this time I did not suspect any grave lesion of the kidney, and I commenced treating her for chronic cystitis. But in a short time I noticed that while she complained of pain everywhere, it still seemed to focalize in the right lumbar region. I therefore proceeded to make a careful physical examination of that portion of the body, with the following results: The costo-iliac space was tender to pressure, and dull on percussion, and I could map out quite distinctly an irregularly roundish tumor, which seemed to be about twice as large as a normal kidney. There was also some tenderness along the course of the right ureter, and pressure upon both the kidney and the ureter seemed to exasperate the bladder and intensify the desire to urinate. A few days later I learned that there seemed to be an intermittent flow of pus; that when the amount of pus was at its maximum the pain in the right side was complained of the least, but whenever the amount of pus in the urine began to diminish, the pain in the right kidney was increased *pro rata*, and all other symptoms were intensified. I now concluded that the patient had what is known as *intermitting pyonephrosis*, and that the symptoms of the renal lesion had been masked by the much more prominent and urgent symptoms emanating from the bladder. I also concluded that the only promise of permanent relief was by means of an operation upon the diseased kidney. With that end in view, I sent her to St. Luke's Hospital, where (it being a charity case), she passed under the care of my colleague, Professor John E. Owens. We examined the case together, and arrived at the concurrent opinion that an operation was urgently demanded, and decided to operate at once. The operation was accordingly performed by Professor Owens, assisted by Professor Gunn and myself, and in the presence of several other members of the hospital staff.

The initial incision was made along the outer border of the erector spinæ muscle, and extended from the iliac crest to the lower border of the last rib. No difficulty was experienced in reaching the kidney, which upon being exposed was found excavated and occupied by many pus-cavities, so that it was practically a multiple abscess. The surrounding tissues were not very much indurated, and there were no adhesions which rendered ablation of the kidney impracticable. But as the operation of nephrectomy was then somewhat new and untried, at least, among our local surgeons, it was thought best to open the kidney upon its dorsal aspect, irrigate it thoroughly, establish free drainage, and give it the best chance to repair itself by granulation. At the suggestion of Professor Gunn the renal cavity was packed with crystals of boric acid, after the proper insertion of the drainage-tube. The wound was then brought together with sutures and dressed antiseptically, and was irrigated daily with a five-per-centum solution of carbolic acid. There was a continuous discharge of pus, which at first was thick and grumous, but afterward became laudable. The amount of pus in the urine gradually diminished, until it got to be hardly noticeable. The patient gained strength, increased in weight, and was out of bed within three weeks after the operation. She left the hospital, contrary to the advice of her medical attendants, about six weeks after the operation. At that time the wound had not entirely healed, but was granulating nicely, and bid fair to close up entirely in a few days.

About two weeks after she left the hospital, she very unwillingly went to ride with her husband. They took a long drive on the lake shore, were caught in a violent storm, and she returned home thoroughly exhausted, chilled, and wet to the skin. The following day she was seized with a severe rigor, followed by an accession of fever, scanty urine, which was tinged with blood and loaded with albumen, œdema of the face and lower extremities, and slight indications of coma. In spite of treatment, these symptoms rapidly grew worse, and she died of *acute nephritis* on the fifth or sixth day after the fatal ride.

*Necropsy*, twenty-four hours after death: Body fairly well nourished; *rigor mortis* fully developed; brain and spinal cord not examined; heart, lungs, and large vessels normal. Liver slightly enlarged, otherwise normal. Stomach and alimentary canal in healthy condition. Uterus and its appendages healthy. The right kidney was shrunken to a mere shell, averaging a little more than a quarter of an inch in thickness; its walls were very irregular, however, measuring in some places more than half an inch in thickness, while in other places nothing was left but the thickened capsule.

There were no pus-pockets found in the organ, but a few small-sized masses of "mortar-like" material, which probably occupied the sites of former pus-cavities. It was impossible to distinguish the different structures of the kidney, but as nearly as we could judge the cortical substance was nearly, or quite, destroyed. The kidney was pretty firmly imbedded in a growth of newly formed connective-tissue, but it could be enucleated without much trouble.

The ureter was funnel shaped, with the mouth of the funnel upward, and was much dilated throughout its full extent; its walls were thickened, and the tube was much longer than normal. Upon laying it open we found several patches of ulceration of its lining membrane. The bladder was very much contracted; indeed it seemed to be in a state of permanent spasm; its cavity would not contain more than two fluid ounces of urine, and even that quantity produced a degree of distension which would have been painful during life. The walls of the bladder were very greatly thickened, the mucous membrane rugose, and the sulci between the corrugations were deeply ulcerated. The urethra was thickened, contracted, puckered into longitudinal folds, and ulcerated.

Upon looking over the history of this case, I am impressed with the idea that it was an exceedingly favorable one for operative treatment, yet the patient died. But the circumstances were mainly against us. In the first place, the operation was not performed as soon as it ought to have been by, perhaps, a couple of years; hence, when it was performed the ureter and bladder were practically destroyed. In the second place, she left the hospital at least two months too soon, and then contracted *acute nephritis* by unwise exposure. I am persuaded that an earlier operation, and perhaps a more radical one, supplemented by absolute control of the patient for a sufficient length of time afterward, would have resulted in a permanent cure. In fact the immediate results of the operation were all that could be expected, and the patient was slowly approaching perfect recovery when she encountered the undue exposure which induced her fatal illness.

CASE II.—On the 8th of July, 1885, I was called to see M. W. P. in consultation with Dr. Dewey, his attending physician. The patient was a remarkably well developed and athletic young man, a lawyer, aged 29. He had always been healthy and strong, and much given to athletic sports. During his college days he frequently engaged in gymnastic exercises, and was the champion athlete of his class. He took particular delight in the "spring-bar" exercise, which calls for violent efforts on the part of the lumbar and dorsal muscles, as well as those of the upper extremities. During one of his periods of exercise, he felt a sudden "strain" in the right lumbar region, more especially in the costo-iliac space. Shortly after, he began to have paroxysmal attacks of pain in this region, not very violent or frequent at first, but gradually increasing in both violence and frequency. He had experienced two or three attacks of great severity, so that on each occasion he was obliged to abandon business and take to his bed. At the time of my visit, he had just returned from a journey of three or four hundred miles. He was attacked while away and was in constant pain on the homeward journey, and went from the sleeping-car to his bed. I found him with a temperature varying from 99° to 101° Fahr. from day to day, with a clammy tongue, impaired appetite, constipated bowels, and with a well-defined area of tenderness over the region of the right kidney, where he also suffered a nearly constant, dull, heavy pain. I failed to detect any tumefaction of the kidney by palpation or percussion, but bimanual pressure (by grasping the loin between the two hands) developed an area of well-defined tenderness. At this time no fluctuation could be detected. I examined the urine with the following results: Specific gravity, 1.018; reaction slightly acid; albumen, a slight cloud, hardly enough for volumetric measurment; sugar, none. The urine was cloudy with mucus, and rather paler than natural. The microscope showed renal and pelvic epithelia in large numbers, isolated and in patches, especially the linear patches or cylinders from the papillary portions of the straight tubes so common in inflammation of the renal pelvis. I also found a great number of pus-corpuscles, some granular matter, but no tube-casts. I concluded that the case was one of suppurating kidney (pyonephrosis), and that the prognosis was grave. I also stated to the attending physician that an operation would be required. About the middle of August fluctuation became quite distinct over the right kidney, and about two fluid ounces of thick, malodorous pus was withdrawn by the aspirator. From this time forward, the patient's condition grew steadily worse; the pain increased; the appetite failed; the tongue became heavily coated; he lost flesh rapidly; night-sweats occurred; the area of tenderness extended, and the whole aspect of the case was discouraging and alarming. Meantime the patient had been treated according to the varying indications, but without any successful results. Having made up my own mind that an operation was unavoidable, I asked that Professor John E. Owens be called in consultation, which was done on September 16. Professor Owens fully agreed with me as to the necessity for prompt operative measures. Accordingly, on the 22d of September, I operated, with the assistance of Professor Owens, Dr. Frank Cary, Dr. Dewey, and Dr. J. H. Bartlett. The line of incision was nearly vertical, along the outer border of the erector spinæ muscle; the kidney was reached without dificulty, and was found firmly united by adhesions to the peri-renal tissues, the latter being much condensed and indurated. The renal cortex was thinned by prolonged suppuration

—in fact, at the point where my dissection reached it, it was but a mere shell of condensed cortical tissue. Upon incising the kidney, there was an escape of thick fœtid pus, perhaps half an ounce in quantity. A free incision was now made through the cortex with a blunt bistoury, using the index finger as a guide. There was but little hæmorrhage, except from a single vessel, but that was easily found and tied. Upon exploring the interior of the kidney with the finger, I found that it was practically *a multiple abscess;* it was beset with pus-pockets in all directions, and these pockets were filled with thick pus, containing gritty masses of urinary salts, and the so-called "mortar-like" matter of Roberts. These cavities I freely opened by perforating them with the finger, and the result was one large ragged cavity in place of many small ones. This cavity was now thoroughly irrigated with a carbolic-acid solution, a large drainage-tube introduced, the wound packed with iodoform-gauze and brought together with deep sutures, except at the lower angle, whence the drainage-tube issued. The patient rallied promptly, and progressed rapidly toward recovery.

The subsequent history of the case is but a simple story of recovery without any untoward incidents.

The temperature never rose above 101.5° Fahr.; the wound healed kindly; the urine was slightly bloody for a few days after the operation, after which it rapidly improved as regards pus and other pathological products; the patient regained his digestion and recovered his strength, and on the 21st of December, following the operation, he went to a distant city to pass the remainder of the winter. On the 20th of April, 1886, he called upon me, having just returned home. He presented the appearance of stalwart health, but complained of some lameness at the seat of the operation, especially if he attempted much muscular exercise. The urine was of normal specific gravity and reaction, but contained a trifle of albumen. There was considerable whitish deposit, consisting of renal, pelvic, and cystic epithelia, leucocytes, mucin casts, and some granular matter, but I found no fibrinous or hyaline casts. These symptoms slowly disappeared; he resumed his business gradually and continuously, and at the present time seems to be in perfect health, the albumen having long been absent from the urine, and the urinary sediment being scarcely noticeable.

Case III.—On the 18th of December, 1886, I was called in consultation by Professor J. H. Salisbury, M. D., to visit Mrs. H., an intelligent lady, who gave me the following history of herself. Her age was forty years; she was married, but had no children; she was a native of this country, and her life had been spent in the ordinary duties of a housewife. Eighteen years prior to my visit the patient had been thrown from a buggy, the seat of which fell upon her right side, fracturing the two lower right ribs. There was intense pain at the seat of injury, but no hæmaturia or other indications of renal injury. In due time there seemed to be complete recovery and entire relief from pain. About seven years after the accident Mrs. H. had a severe attack of pain in the right side, coming on suddenly. The pain was very intense, and required the administration of an anodyne for its relief. This attack was followed by others, occuring once in two or three months, but gradually becoming more frequent and severe. She always located the pain in the right ilio-costal space, and it was accompanied by great tenderness on pressure. With, or, more correctly, following, each attack, there was a partial paresis of the right side, so that locomotion was dificult and painful, and there was a sense of numbness perceptible in the whole right side, including the right half of the tongue. During the paroxysms, respiration was impeded, and sometimes was very difficult and painful. For the past four years these attacks occurred more frequently, and increased in severity and duration, so that her health had been seriously impaired by exhaustion and suffering. They were accompanied, during this latter period, by frequent and sometimes painful urination, and by other sypmtoms of irritable bladder. The urine had long been loaded with pus to such an extent that half a pint of it has been passed with the urine in thirty-six hours. But during the attacks of ilio-costal pain, the pus had either disappeared or diminished to an insignificant quantity, the pain continuing and increasing in severity, and the urine remaining clear until she would suddenly feel something "give way" in her side, the pain would quickly leave, and pus in great quantity would re-appear in the urine. For some hours after the subsidence of the pain, she would experience frequent and urgent calls to urinate, and the fluid evacuated would consist largely of pus. This was the history of the case down to the time I was called by Professor Salisbury, namely, December 18, 1886.

At my first visit I found the patient in bed, in the midst of one of her attacks. She complained of constant heavy, dull pain in the right renal region. Her temperature varied from one to two degrees above normal. She had little appetite, and her digestion was imperfect; she had constipation, followed frequently by diarrhœa; the flow of urine was rather small, but at that time did not seem particularly abnormal to the naked eye. The microscope showed a few pus-corpuscles, epithelia, also, from the kidney and bladder, but no tube-casts. The urine also contained a very small amount of albumen, and an excess of phosphatic salts. Upon examining the patient I found a very distinct tumor in the right ilio-costal space, which extended from the iliac crest to above the level of the last rib, and latterly it extended nearly to the median line. It was tender to the touch, seemed to be rather indistinctly lobulated, gave distinct evidences of fluctuation over a portion of its area, and within certain limits was easily movable. I concluded it was a cystic kidney of the "intermitting" type; that it was much injured by the encroachment of the process of suppuration, and that an operation would be required before any substantial relief could be expected, and this opinion was fully concurred in by Professor Salisbury. A few days after, the accustomed gush of pus came, she was speedily relieved of the constant dull pain, whilst the size of the tumor had diminished.

The patient and her friends finally consented to an operation, and on the 12th of March she entered St. Luke's Hospital for the purpose of having some radical operation performed.

At that time her condition was as follows: She was cheerful; her digestion was good; bowels regular; her sleep was refreshing, and temperature was not above 99.4° Fahr., while the pulse ranged from 80 to 90. She was somewhat emaciated, looked anæmic. Her nervous system was somewhat shattered by the long-continued exhaustion by pain and suppuration. Examination of the urine after her admission into the hospital showed the following results: Specific gravity 1.020; reaction intensely alkaline, so that it took 17 minims of acetic acid (of the U. S. P.) to acidulate ten fluid drachms of urine. The urine remained turbid after filtering from presence of mucus there was a slight amount of albumen present, perhaps a little more than the pus accounted for. The microscope showed crystals of urates of soda and triple phosphates in large amount, disorganized pus-corpuscles in great numbers, and epithelia of the bladder, pelvis of the kidney, and collecting tubes; no tube-casts were found. The tumor in the right ilio-costal space was quite prominent, very easily defined, and somewhat tender upon pressure. Two or three days after her admission, I requested my colleague, Professor John E. Owens, to examine her with me. Upon comparison of our views we fully agreed that an immediate operation was necessary. Accordingly, on the afternoon of the 19th of March, assisted by Professor

Owens, Dr. Frank Cary, Professor Salisbury, Dr. L. B. Hayman, and the house staff, I operated, as follows: An incision was made reaching from the crest of the ilium to the lower rib, a very little to the right of the erector spinæ muscle. The dissection was continued layer by layer until the kidney was reached; it was found surrounded by fatty tissue, which was torn apart by dressing forceps and the fingers. The pelvis of the kidney was found fully distended and the whole organ much enlarged, but there were no adhesions, nor any indications of renal calculi. The organ did not seem to be so much damaged as we expected to find it, and the lesion was mostly located in the pelvic and medullary portion of the kidney, leaving the cortex comparatively unharmed. We therefore thought it best not to attempt the removal of the kidney, but to open the pelvis, irrigate the cavity, and establish drainage, which was accordingly done. An incision about two inches in length was made in the pelvis, the cavity was evacuated of its pus and putty-like matter; the kidney was explored by the finger, but no pus-pockets were found; a drainage-tube was inserted and stitched into the pelvis of the kidney with catgut. The lumbar fascia was next closed by continuous suture of catgut, and the wound then brought together by deep sutures. Reaction came on slowly, but without any bad symptoms, and the patient passed a comfortable night following the operation. For the next few days the current history showed that the patient was restless, nervous, had considerable pain, and required anodynes hypodermically quite frequently, yet she took a good allowance of nourishment, passed a normal amount of urine, and her temperature never rose above 102.2°. The wound was irrigated daily with carbolized water, and the reparative process went on without any difficulty. The urine passed freely through the drainage-tube, but the amount gradually lessened, until after three or four weeks but very little passed that way. The condition of the urine steadily improved, the pus diminishing, the alkalinity lessening, until on the 4th of April, I find the following note: "Urine, specific gravity 1.020; reaction, acid; no albumen; very little sediment." Four weeks after the operation the drainage-tube was removed for the first time. The renal extremity was found coated with urinary salts, showing that it had remained in the renal cavity. Its place was supplied by a tube of smaller size, which was now removed and cleansed every day. The fistulous tract closed in a short time firmly and soundly; the external wound healed rapidly and kindly, and the urine improved daily in its appearance, the amount of sediment lessening, the quantity of urine being about normal, and the general condition of the patient improving in every regard. She remained in the hospital until the 1st day of June, gradually improving in health and slowly gaining flesh during this time. At the date last mentioned she was discharged and went into the country where she spent the summer. I did not see her again until about the 1st of March of the present year, when she called upon me. Her condition had changed marvelously; she had gained in flesh and strength and looks now the picture of health. I procured a specimen of the urine and examined it with the following results: Specific gravity, 1.020; reaction, acid; no albumen; no turbidity from the presence of mucus; very little sediment. Examination of the sediment with the microscope showed a few mucous corpuscles and some epithelium from the bladder and kidney. She states that she has occasionally a little dull, deep-seated pain over the region of the kidney, but it is so slight that it causes her no inconvenience, and probably would not be noticed at all if she were not aware of the fact that there had been in that locality a disease of the kidney. The results of the case may therefore be considered satisfactory in every way.

Case IV.—On the 13th of December, 1887, I was called in consultation by my friend, Dr. J. E. Stubbs, to visit Mr. J. P., a resident of Milwaukee, Wisconsin, but staying at a hotel in this city.

The history of the case was rather meagre, but I was able to obtain the following particulars:

The patient's age was thirty-four years; height, about five feet and eight inches; weight, 155 pounds. He had always been temperate; had never used tobacco to excess, and had never had any venereal diseases. He had lived an active, driving, mercantile life, which involved a great deal of travel and exposure. When he was eleven years old he fell down a rough and precipitous hill-side and received a number of contusions thereby. As a consequence of this he was confined to bed for a few days, and although he remembered being "sore and lame all over," he did not recall any symptoms of renal or urinary trouble. When he was eighteen years old he had "some kind of a fever," but could not tell what. In the autumn of 1883, he was suddenly taken with a severe pain over the right kidney, which followed down the course of the ureter and invaded the urethra and the testicles. He had rigors, a high temperature, persistent pain, and tenderness over the right kidney, and hæmaturia. These symptoms lasted a few days, and confined him to bed, but they gradually improved, and in course of a fortnight he was able to resume his business again. Since that time, however, he has been subject to oft-recurring attacks of pain in the right side, but not of sufficient violence to send him to bed, except possibly on one or two occasions. He had a severe attack of measles last June; the urine was not examined at that time.

He arrived in Chicago three or four days before I saw him. On the morning following his arrival he walked from his hotel nearly a mile to breakfast with some friends. He partook heartily of food, and after some time spent in social intercourse with his friends, started to walk back to his hotel. On his way, he was suddenly seized with a severe paroxysm of pain in the right illio-costal space, so that he was obliged to hail a cab and ride to his hotel. When, two days later, I examined him in conjunction with Dr. Stubbs, I found him with a temperature of 103° Fahr.; pulse 108; dry tongue; thirst and anorexia, and complaining of great pain and tenderness in the right hypochondrium, which extended through the side to the lumbar region. Upon making a physical examination, I found a distinctly rounded tumor, completely filling the right illio-costal space, and extending forward and inward to the median line. The tumor was spherical, quite indistinctly lobulated, tender upon pressure, easily movable within well-defined but narrow limits. Although it was very tense, a distinct feeling of fluctuation could be perceived. It extended upward to the liver, of which it seemed to be a part; below, it reached lower than the crest of the ilium; anteriorly it could be distinctly felt as far as the linea alba, and by making deep pressure fluctuation could be recognized posteriorly at the border of the erector spinæ muscle. Upon examining the urine I found it loaded with pus and alkaline phosphates, and containing a little albumen (about one-fortieth by volume); it was intensely alkaline and offensively ammoniacal, and the patient was plagued by frequent and intense demands to urinate. I at once concluded that the patient was suffering from suppurating kidney, in which opinion Dr. Stubbs concurred. I advised that aspiration be performed at once, and that it be followed by nephrotomy, if the aspirator demonstrated the presence of pus. Unfortunately this advice was not followed, although it was vigorously supported by Dr. Stubbs. The operation was delayed until the patient's friends could correspond with his former medical attendant, who could not believe that the patient was in the condition represented by me, and who accordingly counseled delay and further consultation with Dr. R. G. Bogue, who was called on the 17th. After a most careful and painstaking examination, Dr. Bogue fully concurred in the opinion that an immediate operation was not only justified, but imperatively demanded. We therefore separated to meet on the following morning, September 19, at ten o'clock. At that time the patient's temperature was 102° Fahr.; pulse 108; skin moist and clammy, and the stomach irritable, a condition which was in marked and unfavorable contrast with that which he presented on the 13th. The patient was removed from his bed to an operating table and etherized. There were present, and assisting me, Drs. R. G. Bogue, J. E. Stubbs, L. B. Hayman, and Mr. Stubbs. After anæsthesia was complete, I introduced an aspirator-needle about midway between the point of the lower rib and the umbilicus, and withdrew nearly a pint of fœtid pus, which materially reduced the size of the tumor, although distinct fluctuation could still be detected. It was therefore evident that a radical operation must be performed, and that if performed, a fatal result was by no means unlikely. But after fully explaining to the patient's friends the prospects and dangers involved, it was decided that the patient was entitled to the chances which an operation promised, in spite of its dangers. I accordingly operated, assisted by Drs. Stubbs, Bogue, and Hayman. The initial incision was made about one inch posterior to the anterior superior spinous process of the ilium, extending horizontally upwards to the lower rib. The succeeding muscular and membranous layers were divided as rapidly as possible, but I was considerably hindered and embarrassed by hæmorrhage from the divided planes of muscle. Upon reaching the tumor it was found to be traversed by large veins, which formed a thick plexus all over its surface; the part exposed was tense and fluctuating, showing that the aspirating needle had not penetrated it. An exploring needle was passed into the tumor, which immediately gave exit to a drop of pus. Fearing hæmorrhage if I made an incision into the kidney with a scalpel, the points of a pair of dressing forceps were thrust into the tumor, and I then enlarged the wound a little by inserting a pair of hæmostatic forceps, and forcibly dilating them; this was followed by the insertion of the tip of the index finger, and then of the entire finger. Lastly, the opening was fully dilated by means of the two thumbs placed together, as in dilating the sphincter ani. There was no hæmorrhage of any account, but a gush of not less than a pint of pus. I proceeded next to explore the cavity which had been emptied, and found the "shell" of a kidney enormously enlarged and dilated, but divided into many compartments by dissepiments of pre-existing connective-tissue, answering to the original division into "pyramids of Malpighi." These partitions I tore away as far as I safely could, so as to leave one large cavity. It was my intention to curette the abscess cavity, but the condition of the patient, and the adverse opinion of Dr. Bogue, led me to abandon that measure. The cavity was therefore freely irrigated with a carbolized solution, a large drainage-tube inserted, antiseptic dressings applied, and the patient put to bed in a greatly exhausted condition. Artificial heat and stimulants were vigorously employed; the patient slowly rallied for a few hours, and then without loss of blood or other appreciable cause, he began to sink, and death followed twenty hours after the operation. An examination of the body was not permitted.

It was a sad misfortune that the patient and his friends would not permit prompt measures. I am strongly of the opinion that had the operation been done at once, as Dr. Stubbs and I advised, the patient would have survived it; but the succeeding four or five days of hectic and vomiting were fearfully exhausting, and the result was just what might be expected.

I would not again attempt to reach the kidney by the route traversed in this case, not even if I were performing nephrectomy. But for nephrotomy, the lumbar incision, by the side of the erector spinæ muscle, is infinitely preferable for many reasons, which I purpose formulating in a future article.

Lastly, I desire to extend my sincere thanks to Dr. Bogue for his most valuable counsel and assistance.

